# Selection Against Hybrids Maintains Genetic Divergence Between Populations of a Coastal Cleaner Fish Translocated Across a Genetic Break

**DOI:** 10.1111/eva.70214

**Published:** 2026-03-15

**Authors:** Enrique Blanco Gonzalez, Per Erik Jorde, Sissel Jentoft, Joana Isabel Robalo, Mana Naito

**Affiliations:** ^1^ Department of Natural Sciences University of Agder Kristiansand Norway; ^2^ Centre for Coastal Research University of Agder Kristiansand Norway; ^3^ Institute of Marine Research, Flødevigen His Norway; ^4^ Department of Biosciences, Centre for Ecological and Evolutionary Synthesis University of Oslo Oslo Norway; ^5^ MARE ‐ Marine and Environmental Sciences Centre, ISPA Instituto Universitário de Ciências Psicológicas, Sociais e da Vida Lisboa Portugal

**Keywords:** corkwing wrasse, fisheries enhancement, mating behavior, reproductive fitness, sexual selection, *Symphodus melops*

## Abstract

Genetic divergence between populations and/or species is mainly driven by reproductive isolation, that is, reduced gene flow, due to either pre‐ and/or postzygotic barriers. In this study, we investigate the role of (i) reproductive ecological interactions and (ii) mechanisms of evolutionary isolation between two genetically differentiated populations of a coastal fish species, corkwing wrasse *Symphodus melops* , in a large semi‐natural mesocosm basin over a 2‐year period. Corkwing wrasse is one of the main cleaner fish species used by the salmon aquaculture industry in Europe, where translocation is a common practice. The parentage analysis performed on the group of offspring survivors at the end of the translocation experiment evidenced strong selective winter mortalities in hybrid offspring (with mixed parental origin). Additionally, it suggested unprecedented assortative mating patterns in corkwing wrasse. Fish of west Norwegian origin experienced stronger intensity of sexual selection and displayed higher relative reproductive success (RRS) than individuals of southern origin under the experimental conditions. Size of the breeder and alternative sneaking behavior in males are two phenotypic traits often selected to attract potential mates and maximize reproductive fitness. However, neither of them had a significant effect on offspring production. Hence, our results suggest that other traits related to mate choice and competition, not examined here, could play a major role. Our results revealed strong selection against hybrids and potential assortative mating between southern and western corkwing wrasses population as two major contemporary drivers reshaping the historical genetic divergence associated to founder events during post‐glacial recolonization in Norway. We discuss the relevance of our findings for aquaculture‐based fisheries enhancement systems, resource management and conservation.

## 1 Introduction

Sexual selection is a form of selection which favors traits that increase mating success and contributes to explain the behavioral and morphological differences commonly observed between sexes of a particular species (Andersson 1994). Successful mating involves behavioral and genetic interactions between individuals of both sexes to compete for access to mates, that is, intrasexual selection, or to choose mates of the opposite sex, that is, intersexual selection (Alonzo 2010; Coleman and Jones 2011). Population density and sex ratio will determine intrasexual competition as the strength of sexual selection is expected to be larger for the sex over‐represented when access to mates is limited. They will also experience higher variance in mating success and reproductive success. At the same time, individuals of the sex under‐represented will have the possibility to choose among mates that predict the most beneficial matings (Jones 2009; Alonzo 2010; Aronsen et al. 2013). The slope of the relationship between mating success and reproductive success, the Bateman gradient *β*_*ss*_, is also expected to be steeper in the sex over‐represented (Bateman 1948; Arnold and Duvall 1994; Collet et al. 2014).

Fishes display the whole spectrum of mating systems, and the spatial and temporal availability of resources and/or mates are important variables for mate monopolization and the intensity of sexual selection (Emlen and Oring 1977; Avise et al. 2002; Taborsky et al. 2008; Alonzo 2010; Coleman and Jones 2011). Indeed, fishes have evolved a variety of alternative reproductive tactics to successfully mate with several individuals of the opposite sex and maximize their reproductive output. In some species of salmonids and wrasses, for example, it is common to invest energy into protecting a territory or constructing and guarding nests from competitors. These species often display remarkable differences in traits selected to attract potential mates and maximize fitness, such as size dimorphism, color polymorphism, or sneaking behavior (Uglem et al. 2000; Avise et al. 2002; Serbezov et al. 2010; Alonzo 2012; Halvorsen et al. 2016). Competition for the best territories to build and guard nests anticipates reproductive fitness advantage by attracting visitors of the opposite sex. At the same time, female promiscuity and sexual selection on males are predicted to reduce energy investment in male care (Andersson 1994; Jones and Ratterman 2009). However, the increasing number of recent studies deviating from the traditional theoretical models advocates for reconsideration of the inter‐ and intra‐sexual interactions involved in mating and parental care in fish (Alonzo 2010; Coleman and Jones 2011).

Nest building with parental care behavior in fishes is often under both natural and sexual selection (reviewed by Svensson and Kvarnemo 2023). While choosing a good nest‐builder who locates a well‐built nest in the appropriate location is under natural selection, the fact that such behavior may attract and facilitate access to individuals of the opposite sex is also under sexual selection (Alonzo 2010; Bose et al. 2018; Svensson and Kvarnemo 2023). Populations adapted to different environmental conditions can also exhibit differential sexual selection (Servedio and Boughman 2017; Svensson and Kvarnemo 2023). The intriguing interplay between natural and sexual selection may counteract their individual effects, although they often work in harmony to promote reproductive isolation (Svensson et al. 2018; Perini et al. 2020; Labonne et al. 2021; Svensson and Kvarnemo 2023). Human‐driven perturbations such as size‐ and sex‐selective fishing can also induce evolutionary adaptations in life history traits. Intensive fishing can affect the size, age, and sex structure of populations and cause changes in traits involved in mating choices and sexual selection (Halvorsen et al. 2016; Sbragaglia et al. 2019; Matte et al. 2023). Common garden and transplantation experiments offer the possibility to study these complex interactions and gain insights into mating systems and the evolutionary and ecological dynamics of species and/or populations (Vines and Schluter 2006; Blanquart et al. 2013; Connallon et al. 2018; Svensson et al. 2018; Perini et al. 2020; Labonne et al. 2021).

Corkwing wrasse, *Symphodus melops* , is a rocky shore fish species heavily targeted as cleaner fish to remove sea lice infestation by the salmon aquaculture industry in Europe. The species displays sexual size dimorphism and males present two alternative reproductive behaviors fixed throughout their lifespans. Large territorial males build nests and provide parental care against smaller sneaker males, which mimic female phenotypes, mature faster and attain smaller body sizes than territorial males (Uglem et al. 2000; Halvorsen et al. 2016). The Norwegian wrasse fishery is size‐ and sex‐selective, particularly interested in large territorial males (Blanco Gonzalez and de Boer 2017). Individuals inhabiting the warmer southern and colder western part of the country were found to present significant phenotypic and genotypic differences (Knutsen et al. 2013; Blanco Gonzalez et al. 2016; Halvorsen et al. 2016); yet millions of fish are translocated annually to salmon farms located in the west coast (Blanco Gonzalez and de Boer 2017). Previous genetic studies reported the presence of a genetic break and reproductive isolation between southern and western wrasses associated to historical founder events during the colonization of the Scandinavian Peninsula and the presence of unsuitable sandy habitats (Blanco Gonzalez et al. 2016; Mattingsdal et al. 2020). Results of whole genome resequencing evidenced ongoing gene flow between the two populations across the break and suggested recent contact or negative selection against hybrid (Mattingsdal et al. 2020). Similarly to other temperate rocky shore fish species at the cold edge of their distribution ranges, corkwing wrasse enter a hypometabolic state and experience high mortalities (i.e., natural selection) during the cold winter periods in Norway (Bjelland et al. 1996; Blanco Gonzalez and de Boer 2017). Poor sea lice cleaner performance and high mortalities during the cold months are of major concern to the salmon industry.

In a translocation experiment performed in a large seminatural mesocosm basin, Blanco Gonzalez et al. (2019, hereafter BG2019) found high levels of interbreeding between locally (southern) caught and translocated (western) breeders and a higher proportion of juveniles from the latter compared to the former group of breeders. While that study unveiled important information on the reproductive biology of the species, relevant for management and conservation, the group of offspring analyzed (651) only represented a small proportion of few‐months‐old fish collected in the mesocosm during summer, after the spawning season.

Here, we followed up the same cohort of corkwing wrasse born in the experimental mesocosm previously described by BG2019 and performed a parentage analysis on all offspring that survived the following harsh winter period, when the water in the upper layer of the basin froze. Using parental relationships, it is possible to disentangle sexual and natural selection during the early developmental stages and the juvenile phase and, at the same time, gain insight into the selective effects of cold‐water temperature on the survival of naturally born individuals of local, translocated, and hybrid parental origin. We further assessed the consistency in patterns of family composition before and after the cold winter period and expanded on the knowledge on reproductive behavior gained in the previous work (BG2019). Current findings also provide insights into mating patterns and evolutionary mechanisms in a coastal marine fish of relevance for aquaculture‐based fisheries enhancement, resource management, and conservation.

## 2 Material and Methods

### 2.1 Study Species and Experimental Setting

**FIGURE 1:**
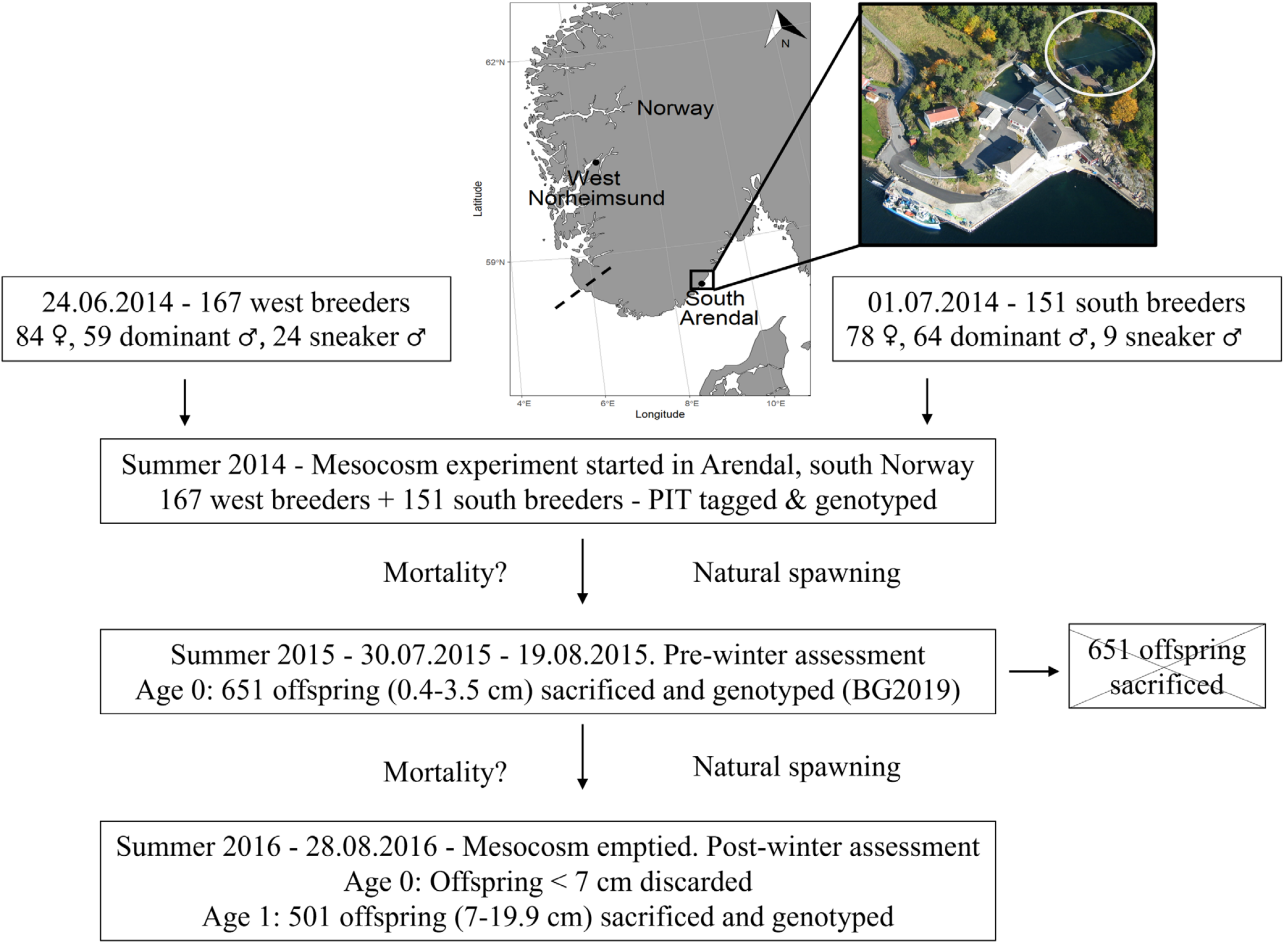
Overview of the structure and experimental set up of the study. The map shows the geographical locations where the breeders of west (Norheimsund) and south origin (Arendal) were collected. The white circle in the aerial photo illustrates the mesocosm basin where the experiments were conducted at the research facilities of the Institute of Marine Research in Arendal, Norway. The dashed line represents the approximate location of the genetic break separating south and west corkwing wrasse populations in Norway (Blanco Gonzalez et al. 2016).

This study was performed at the research facilities of the Institute of Marine Research at Flødevigen in Arendal, on the south coast of Norway. Figure 1 provides an overview of the complete experimental set up. The groups of breeders of western (Norheimsund, 167 specimens) and southern (Arendal, 151 specimens) origin were transported alive and introduced into the mesocosm basin at Flødevigen on June 24 and July 1, 2014, respectively. This period coincides with the spawning season of corkwing wrasse in Norway (Blanco Gonzalez and de Boer 2017; Uglem et al. 2000). After arrival, the total length (cm), weight (g), and sex of each breeder were determined by examining the urogenital papilla (only present in females and sneaker males) and inspecting a small ejaculate sample of eggs/sperm obtained by gently massaging the abdomen of the fish. Each parental fish was finclipped for microsatellite DNA parental analysis and individually tagged by implanting a Passive Integrated Transponder (PIT) tag for individual identification. Further details on the collection of the parental breeders and characteristics of the experimental mesocosm basin are provided in BG2019. In that study, a group of few‐months‐old juveniles (651 specimens) were collected from the mesocosm at the end of the spawning season in summer 2015. The offspring were sacrificed and finclipped for microsatellite DNA analysis to investigate the parental contribution before the cold winter period. The experiments in the mesocosm continued for an additional year, until August 28, 2016, when the mesocosm basin was emptied and all remaining fish were collected. Survivor fish were scanned to detect the putative presence of previously PIT‐tagged breeders. The group of offspring was measured in total length (cm) and weighed (g), and those fish smaller than 7 cm were excluded from the analysis as they likely represent age‐0 juveniles born during summer 2016 (Halvorsen et al. 2016). Therefore, the group of 501 offspring used in this study comprises fish born in the mesocosm in summer 2015, ranging between 7 and 19.9 cm in total length (Table 1). A small fin tissue was clipped from the 501 offspring survivors collected from the mesocosm and stored in 95% ethanol for DNA analysis.

**TABLE 1:** Summary of corkwing wrasse samples and statistics of genetic variability at 10 microsatellite markers.

Sample ID	Collection	*N*	Sex	TL	BW	*A*_r_	*H*_O_	*H*_E_	*F*_IS_
Breeder south	24.06.2014	151	78:64:9	10.5–20.5	14.6–108.0	7.39	0.668	0.658	0.015
Breeder west	01.07.2014	167	84:59:24	10.5–19.5	16.4–101.7	8.73	0.761	0.738	−0.032
Offspring south	28.08.2016	21		8.4–18.8	26.4–91.6	5.98	0.633	0.6617	0.043
Offspring hybrid	28.08.2016	86		7.3–19.3	18.3–92.7	6.75	0.810	0.717	−0.131
Offspring west	28.08.2016	393		7.0–19.9	14.1–108.1	7.02	0.741	0.717	−0.033

*Note:* Sample ID, collection date, *n* = sample size, sex (number of females:nesting; males:sneaker males), TL = total length range in cm, BW = body weight range in g, average over loci allelic richness (*A*_r_), observed (*H*_O_) and expected heterozygosity (*H*_E_), and deviation from HWE (*F*_IS_).

### 2.2 Genetic Analysis and Pedigree Reconstruction

Total genomic DNA extraction and individual genotyping analyses were conducted on all 501 offspring survivors using the same eleven polymorphic microsatellites and multiplex PCR procedure as described in BG2019. Microsatellite genotyping was performed in the genetic laboratory of the Institute of Marine Research at Flødevigen in Arendal using the same ABI 3130XL automatic genetic analyser, size standard and bins for individual genotyping with GENEMAPPER v. 4.0 (Applied Biosystems) as in the previous study. As routine in the lab to safeguard against potential genotyping error, approximately 5% of the samples were randomly subjected to repeated genotyping, including some of the parental fish genotyped in BG2019. However, individual genotyping at locus SMB118 was only used to resolve parentage assignment for a few “ambiguous” offspring allocated by PAPA (below) because the presence of null alleles at this locus was previously suspected (for details, see BG2019). Therefore, further analysis was limited to 10 microsatellite markers. Genetic variation for the samples, southern and western breeders and the offspring, was determined by calculating allelic richness (*A*_r_), observed (*H*_O_), and expected heterozygosity (*H*_E_), and total gene diversity over all samples (*H*_T_) based on Nei and Chesser (1983), using FSTAT v.2.9.3.2. (Goudet 1995). We estimated *F*_IS_ based on Weir and Cockerham (1984) and tested for deviations from Hardy–Weinberg (HW) equilibrium by the Markov chain procedure with 100,000 dememorization steps, 1000 batches, and 50,000 iterations per batch with GENEPOP package v.1.1.7. (Rousset 2008) in R (R Core Team 2023). The false discovery rate (FDR) approach (Benjamini and Hochberg 1995) at the 5% level was adopted with the *p*.adjust function in R when interpreting the significance of *p* values in situations of multiple tests. Linkage disequilibrium (LD) among locus pairs within each sample was tested for by a *G*‐test with 10,000 demorizations, 100 batches, and 1000 iterations per batch in GENEPOP v.1.1.7 (Rousset 2008). MICROCHECKER v.2.2.1 (van Oosterhout et al. 2004) was used to investigate the presence of null alleles, stutter‐bands or other technical artifacts. The discrimination power of the set of microsatellite loci for parentage analysis was evaluated by the exclusion probability (*Q*) and the probability of identity index (*I*) with Cervus v.3.0 (Kalinowski et al. 2007). We estimated the cumulative success rate of parentage allocation in PARFEX v1.0 (Sekino and Kakehi 2011) by ranking the markers according to the exclusion probability of both parents, Excl. P2 option, based on 1000 offspring simulations. Wright's *F*_ST_, using Weir and Cockerham's (1984) estimator *θ* applied to all samples and between pairs of samples, was estimated to quantify genetic differentiation among samples. The statistical significance of *p* values was examined by *G*‐test in GENEPOP package v.1.1.7 (Rousset 2008) with 100,000 dememorization steps, 1000 batches, and 50,000 iterations per batch. The FDR approach proposed by Benjamini and Yekutieli (2001) was adopted to correct for multiple tests in pairwise tables and *p*‐values were judged for significance at the 5% level. Genetic differentiation between the five groups (two breeders and three offspring) was also examined by transforming the allelic information into components explaining most of the genetic variation using the Principal Components Analysis (PCA) implemented in the Adegenet package (Jombart 2008) in R (R Core Team 2023).

Parentage assignment was performed in PAPA v.2.0 (Duchesne et al. 2002) and corroborated in CERVUS v.3.0 (Kalinowski et al. 2007) following a similar approach as described in BG2019. Nesting males were individually coded Mxxx, sneaker males Sxxx, and females Fxxx. The uniform error rate for the assignment in PAPA was set to 0.02, while the parental inference in CERVUS was done to the breeder pair with the highest log‐likelihood (LOD) score. A major advantage of the current experimental setup lies in the fact that all potential breeders were sampled and genotyped, and their sexes known; hence, the allocation was considered correct only when a trio (offspring and a parental pair) showed no mismatch at any locus, and PAPA and CERVUS showed consistent parental pair assignments. As a measure to prevent genotyping errors and parental‐offspring misassignment, we repeated the assignment test with a maximum mismatch at two loci and revised allele scoring for trios of those offspring not allocated to any parental pair or allocated to more than one pair. In those cases where misassignment persisted, we repeated genotyping at all loci and re‐ran the analysis. One out of 501 offspring could not be unambiguously assigned to a single parental pair, and it was therefore removed from further analysis (detailed in the results section). Based on the results of the parental assignment, offspring were classified as “south” or “west” when both parents were of south or west origin respectively, or “hybrid” when parents were of different origins.

### 2.3 Reproductive Success and Mating Success

The results of the parentage analysis were used to estimate reproductive (RS) and mating success (MS) per breeder, as follows. RS was defined as the total number of offspring produced by each breeder that reached reproductive maturity during the 2‐year experimental period until the mesocosm was emptied. Corkwing wrasse in southern Norway matures within their first or second year (Halvorsen et al. 2016); therefore, a large proportion of the individuals analyzed in this study are presumed to have reached maturity. MS was defined as the total number of breeders with which successful mating took place, that is, number of breeders a fish got offspring with, during the 2‐year experimental period at the mesocosm basin. Mean values of RS and MS were estimated separately for gender type (females, nesting males and sneaker males) as well as for origin (south and west). In addition, we calculated Relative Reproductive Success (RRS) for each gender type by dividing the average RS of non‐local west fish by the average RS of local south fish of the same gender. We also estimated RS for breeder pairs and estimated RRS for non‐local west and hybrid breeder pairs by dividing their average RS by the average RS of local south breeder fish. The statistical significance of RS comparisons was determined by ANOVA tests and RRS tests were conducted by permutation tests in R (R Core Team 2023).

Additionally, we estimated the inbreeding effective number of breeders, *Nb* (Crow and Kimura 1970; Waples 2002), with and without taking parental origin into consideration as:

$$Nb_{f,m}=\frac{\overset{ˊ}{k}N-2}{\overset{ˊ}{k}-1+\frac{\mathrm{Vk}}{\overset{ˊ}{k}}}$$

where *N* represents the number of breeders, $\overset{ˊ}{k}$ the average number of offspring of year 2016 cohort and *V*_k_ the variance in the number of offspring from contributing breeders. Values were calculated separately for male (m) and female (f) parents and combined to obtain the inbreeding effective number of breeders

$$\mathrm{Nb}=\frac{4*{\mathrm{Nb}}_{f}*{\mathrm{Nb}}_{m}}{{\mathrm{Nb}}_{f}+{\mathrm{Nb}}_{m}}$$

Assortative mating among breeders according to origin (south and west) and sex (male and female) was tested by a 2 × 2 contingency table with Yates correction. As only a few individuals displayed sneaking behavior, all males were grouped together regardless of their mating behavior (sneaking or nesting). To further understand the possible causes explaining the variance in reproductive success, we used generalized linear models (GLMs) that included parental origin (south and west) of males and females, whether both parents were of the same origin or not, length of males and females, and male behavior (nesting males and sneaker males) as variables in the models.

RS ~ Male origin + Female origin + Both parents of same origin + Male length + Female length + Male behavior.

Since the number of offspring recovered from the mesocosm (501 fish) was very small in relation to all possible breeding pairs combinations (potentially 156 male and 162 female parents, yielding 25,272 pair combinations), we first investigated the effect of the variables on the success/failure to produce offspring. We used a negative binomial distribution with a log link function in R (R Core Team 2023) and the coefficients of the model were estimated using maximum likelihood estimation. We further investigated the effect of the same variables on the number of offspring produced with a second GLM with a Poison distributed response variable. In this case, the analysis was performed only including successful breeding pairs that produced at least one sampled offspring.

## 3 Sexual Selection

To characterize the mating system and measure sexual selection we determined several metrics based on Bateman's three principals (Bateman 1948; Arnold 1994; Collet et al. 2014). Following the approach by Lande and Arnold (1983), reproductive success and mating success were standardized to have a mean of 0 and a standard deviation of 1. Standardization of the total length of the fish was performed by subtracting the trait mean and dividing by the trait standard deviation for each fish. The opportunity for selection (*I*) was estimated as the variance in relative reproductive success while the opportunity for sexual selection (*I*_*s*_) was estimated as the variance in relative mating success. The standardized selection differential (*s*) was estimated as the covariance between trait values and relative reproductive success, while the standardized mating differential (*m*) was estimated as the covariance between trait values and relative mating success. The Bateman gradient (*β*_*ss*_) was calculated as the slope of the least regression of relative reproductive success on relative mating. We also determined the upper limit on the intensity of selection on the trait by the maximum standardized sexual selection differential (*s*_*max*_) proposed by Jones (2009) as *β*_*ss*_ × $\sqrt{\mathrm{Iₛ}}$. Our estimates include individuals without any offspring. Although this approach may overestimate the potential strength of sexual selection (Arnold and Duvall 1994), they represent an important component of the variance in fitness (Wade and Shuster 2004). Estimates were determined separately by origin (south and west) and gender (females, nesting and sneaker males) and tested for significances with ANOVAs.

## 4 Results

### 4.1 Genetic Diversity

The complete genotyping of 819 fish (318 potential breeders and 501 offspring) at ten polymorphic microsatellites scored 175 alleles (ranging from 4 to 40) and overall total gene diversity, *H*_T_, was 0.731 (ranging from 0.519 to 0.908, Supporting Information, Table S1). Overall estimate of genetic divergence evidenced significant differences between the southern and western breeders and the offspring (*F*_ST_ = 0.059, *p* < 0.05). Considering each locus separately, *F*_ST_ estimates ranged between 0.129 and 0.004, and allele frequencies among samples differed significantly at all loci (Table S1). The highest overall levels of genetic variability were found in the broodstock from the west coast (*A*_r_ = 8.73 and *H*_E_ = 0.738, Table 1). Pairwise *F*_ST_ estimates evidenced significant differences (at the 5% level after the FDR) between each sample pair, except the breeders and offspring of west origin (*F*_ST_ = −0.001 *p* < 0.555, Table S2). These patterns of genetic differentiation appear corroborated in the PCA analysis (Figure S1) where breeders and offsprings of the same origin clustered close to each other and far apart from those individuals of different origin. Meanwhile, hybrid offspring are centered between individuals of the two origins (south and west) along PC 1 (c.f. Figure S1). After the FDR correction (at the 5% level), nine of the 50 possible locus‐sample combinations evidenced deviations from Hardy–Weinberg expectations (*F*_IS_). All cases were attributed to an excess of heterozygotes in the offspring samples. Similarly, after the FDR correction (at the 5% level), 58 of the 225 possible pairwise tests of LD were found statistically significant. All cases were detected in the offspring samples: 40, 17, and 1 cases in west, hybrid and south offspring, respectively. The results provide no reason to conclude that the loci were linked.

## 5 Reproductive Success and Mating Success

The set of microsatellites used in this study displayed strong power for parental assignment (Supporting Information Table S1) and PARFEX (Sekino and Kakehi 2011) estimated a 100% accumulative success rate of parentage allocation ranking markers on exclusion probability of both parents, Excl. P2 (data not shown). The parental pair of one (out of 501) offspring could not be unambiguously assigned to a single parental pair, and it was therefore removed from further analysis.

**FIGURE 2:**
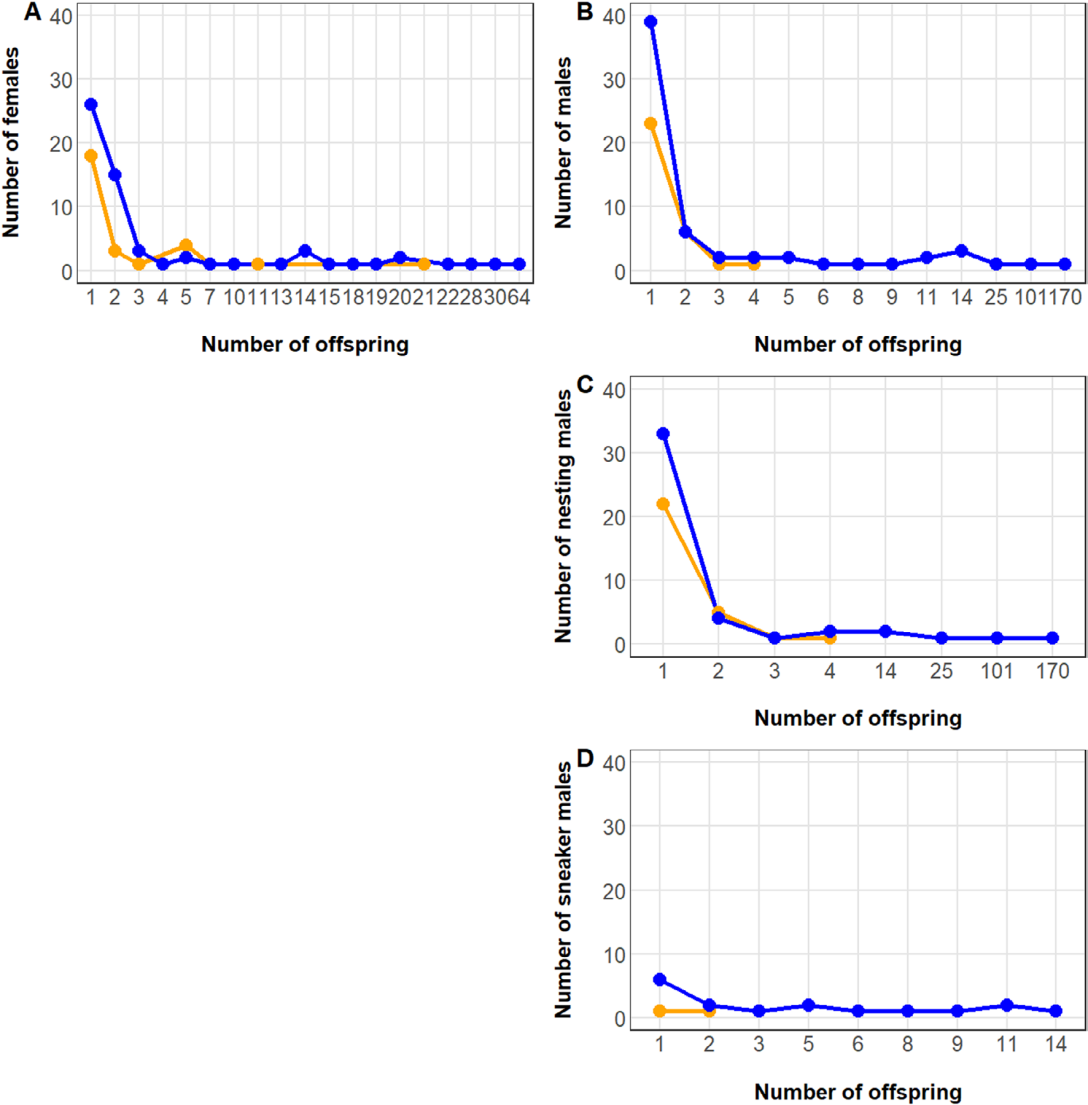
Cumulative curves of reproductive success (i.e., number of offspring produced) by females (A), all males combining nesting and sneaker males (B), only considering territorial males (C) and only considering sneaker males (D). Blue (South) and orange (West) colors represent the origin of the breeder. Note the discontinuity in the *x*‐axis.

The parental assignment of the remaining 500 offspring resulted in 393 west, 86 hybrid and 21 south offspring (Table 2). It revealed the contribution from 93 males (31 of south and 62 of west origin) and 92 females (29 of south and 63 of west origin) (Figure 2), representing 59.6% and 56.7% of the 167 and 151 potential breeders, respectively. Among males, the proportion of nesting: sneaker fish assigned was 29:2 and 45:17 for the southern and western group, respectively. Over half of the offspring were assigned to two large nesting males, M447 and M435, with 170 and 101 fish, respectively. Among females, the greatest number of offspring were assigned to F647, F301 and F341 with 30, 28 and 27 fish, respectively; while S323 and S313, with 14 and 11 offspring, respectively, showed the highest Reproductive Success among sneaker males. All major contributors were fish of non‐local western origin. The largest contributors of southern origin were females F044 and F219 with 21 and 11 offspring, respectively, nesting male M108 with 4 offspring and sneaker male S267 with 2 offspring (Figure 2).

**TABLE 2:** Numbers of offspring of south, hybrid and west origin collected from the mesocosm in 2015 (pre‐winter) and 2016 (post‐winter).

Collection year	South	Hybrid	West	Sum	Source
2015	17	195	438	650	BG 2019
2016	21	86	393	500	This study

**FIGURE 3:**
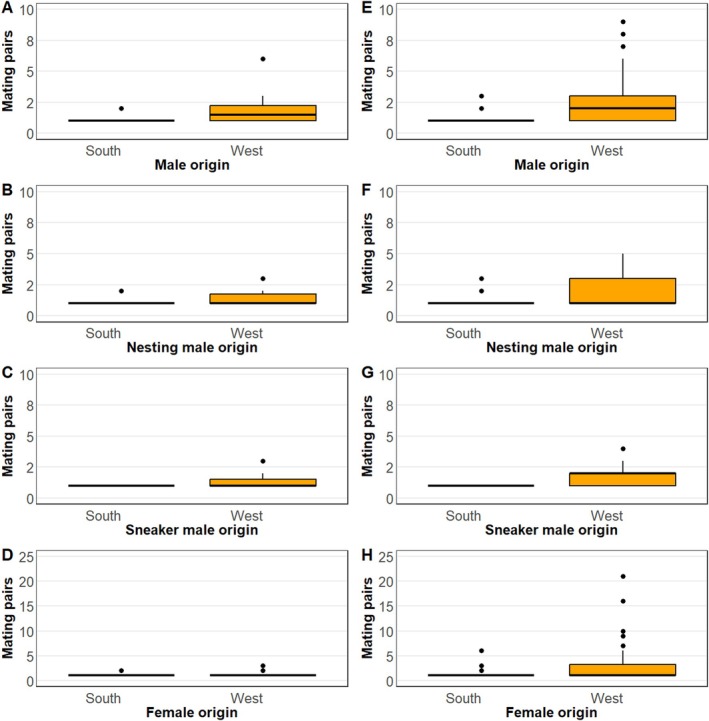
Number of mating pairs according to broodstock origin (South, A–D and West E–H) and sex (Females, A–C and E–G, and Males D and H). The numbers of mating pairs for females are shown combining nesting and sneaker males (A, E), only considering nesting males (B, F) and only considering sneaker males (C, G). Blue (South) and orange (West) colors represent the origin of the mating pair. Note the difference in the scale of the *y*‐axis between sexes.

Our results also evidenced high variability in Mating Success, concerning both origin and gender of the breeders (Table 3, Figure 3, Figure S2). Males producing the largest number of offspring (Figure 2) were also the most successful to mate with multiple partners (Figure S2). Males M447 and M435 successfully mated with 27 and 19 different females respectively while the most successful sneakers (S309 and S323) mated with 8 females. Polygamy was also common among females (Figure 3), with the most successful females (F295, F301, F369, and F405) mating with 9 different males each, Figure S2. Hence, the parentage assignment revealed that corkwing wrasse can display different mating behaviors including monogamy, polygyny, polyandry, and polygynandry (Table 3, Figure 3, Figure S2). A total of 222 different families were identified, most of them (150, 68%) involved both parents of west origin in contrast to only 21 (9%) families where both parents were of south origin. Surprisingly, all mating pairs involving both parents of south origin were present with only a single offspring each, in contrast to the largest western (45 offspring, breeding pair M447‐F369) and hybrid families (14 offspring, M447‐F044) (Figure S2).

**TABLE 3:** Mating system and sexual selection statistics by gender type; in parenthesis are the number of individuals for each type.

Gender type	RS South		RS West		RS combined		RRS
	Mean	Variance	Mean	Variance	Mean	Variance	
Females (*n* = 162)	1.132	8.702	4.814	91.706	3.086	55.868	4.254
Nesting males (*n* = 123)	0.609	0.686	6.373	651.928	3.374	318.646	10.458
Sneaker males (*n* = 33)	0.333	0.500	3.417	17.906	2.576	14.939	10.250

*Note:* RRS (relative reproductive success) for each gender type is the average RS of non‐local west fish divided by the average RS of local south fish of the same gender.

The combined mean RS among gender types ranged between 2.6 in sneaker males and 3.4 in nesting males; however, mean RS was highly skewed when looking at origin and gender separately (Table 3, Figure 2). Among western breeders, mean RS in nesting males was higher (6.373) than in females (4.818) and sneaker males (3.417). In contrast, mean RS in females of south origin almost doubled the estimates of nesting males and triplicated sneaker males (cf. Table 3). For all genders, mean RS in the breeders of west origin were several folds higher than in those from the south, four times in females and over ten times for both nesting and sneaker males (Table 3). A similar pattern was found by looking at mean RS by breeding pairs (Table 4). RRS (i.e., relative RS of non‐local (west or hybrid) compared to local (south) breeders) evidenced high success among non‐local breeders, both within gender (Table 3) as well as breeding pairs (Table 4). The large variance in individual RS among breeders (Tables 3 and 4) led to a low inbreeding effective number of breeders, *Nb*. This effect was particularly noticeable in the breeders of west origin, *Nb* = 16. Although mean RS in the breeders of south origin was several folds lower than that of their western counterparts (Tables 3 and 4), the similarity in family sizes (i.e., lower variance, *Vk*) contributed to a higher overall *Nb* = 36 for them. Combining the results without taking parental origin into account yielded *Nb* = 20. This number represents only 6.29% of the total number (318) of potential parents.

**TABLE 4:** Mating system and sexual selection statistics by breeding pairs; in parenthesis are the number of individuals for each group.

Breeder pair	Reproductive Success		RRS
	Mean	Variance	
Both breeders from the south (*n* = 151)	0.003	0.003	
Breeders of different origin (*n* = 318)	0.007	0.028	1.994
Both breeders from the west (*n* = 167)	0.055	0.580	15.845

*Note:* RRS (relative reproductive success) for non‐local west and hybrid breeder pairs is the average RS divided by the average RS of local south breeder fish.

### 5.1 Factors Affecting Reproductive Success

The abovementioned disparity in MS and RS between gender types appears supported by assortative mating patterns where mating between breeders of south and west origin differed with expectations under random mating (*χ* ^2^ = 20.12, *p* < 0.001, c.f. Table 5). Parameter estimates for the GLM analyses of success of a breeding pair to produce offspring and family sizes are presented in Table 6. The logistic regression analysis inferred that the probability to successfully produce at least one offspring was exp. (−8.170)/(1 + exp. (−8.170)) = 0.00028. This probability increased significantly with the presence of a western breeder. It was tripled in case of a male exp. (−8.170 + 1.154)/(1 + exp. (−8.170 + 1.154)) = 0.00090 and doubled in case of a female breeder exp. (−8.170 + 0.783)/(1 + exp. (−8.170 + 0.783)) = 0.00062. In case both parents were of western origin the estimated probability increased seven‐fold, from 0.00028 to exp. (−8.170 + 1.154 + 0.783)/(1 + exp. (−8.170 + 1.154 + 0.783)) = 0.00196. In contrast, neither size of the breeder or alternative sneaking behavior in males had a significant effect on offspring production. On average, an average‐sized male (12.806 cm) mating with an average‐sized female (13.194 cm) produced 1.05 offspring: exp. (0.328 + (0.032 × 12.806) + (−0.052 × 13.194)) = 1.05. The presence of a male of western origin increased the family size to 2.68 on average, in contrast to the adverse effects of alternative sneaking male behavior which resulted in significantly smaller families, 0.48. On the other hand, the number of offspring produced was not significantly affected by female origin, breeding pair of same origin or breeder body length (Table 6).

**TABLE 5:** Mating patterns, family composition, among breeders according to origin (south and west) and gender type (male and female).

		Females		Total
		South (*n* = 78)	West (*n* = 84)	
Males	South (*n* = 73)	21	19	40
	West (*n* = 83)	32	150	182
	Total	53	169	222

*Note:* In parenthesis are the number of individuals for each group. Contingency chi‐square tests with Yates' continuity correction, *χ* ^2^ = 20.12, df = 1, *p* < 0.001.

**TABLE 6:** Parameter estimates from the logistic regression models on the effects of phenotypic traits of the breeders (total length both for males and females), reproductive behavior (either nesting or sneaker male) and origin (either south or west for both males and females) on mating success (MS, i.e., success/failure on offspring production) and on reproductive success (RS, i.e., number of offspring produced by successfully mated breeding pairs).

Model parameter	MS		RS	
	Estimate	*p*	Estimate	*p*
Intercept	−8.170	**< 0.001**	0.328	0.559
Male origin	1.154	**< 0.001**	0.936	**< 0.001**
Female origin	0.783	**< 0.001**	0.049	0.779
Both parents of same origin	0.838	**< 0.001**	0.081	0.629
Male length	0.050	0.260	0.032	0.270
Female length	0.069	0.086	−0.052	0.057
Sneaker male	0.199	0.199	−0.782	**< 0.001**

*Note:* Bold values represent statistically significant results (*p* < 0.05).

### 5.2 Sexual Selection

To assess the effects of sexual selection on the strong variance in RS and MS observed both by origin and gender type (Tables 3 and 4), we estimated sexual selection parameters for total body length (Table 7). Although the GLM analyses suggested no significant effect of length either on offspring production or family size (Table 6), this phenotypic trait has been found to differ significantly between gender types and geographical regions (west vs. south) in Norwegian corkwing wrasse populations (Halvorsen et al. 2016). Overall, we found (Table 7) that breeders of southern origin displayed larger standardized variance in MS (i.e., *I*_*s*_) than the western ones, while the opposite was true when looking at the standardized variance in RS (i.e., *I*). ANOVA tests on Bateman gradient, *β*_*ss*_, differed significantly from zero for breeders of both south and west origin. The steeper regression slope in the latter group (1.5 times larger) suggests stronger intensity of sexual selection in the breeders of west origin. For both groups, the intensity of selection on length (*s*) only represented 2%–6% of the maximum standardized sexual selection differential, *s*_*max*_ (Table 7). Looking at gender for each origin separately, in the southern group, selection estimate parameters in females were consistently higher than in nesting males, except for *s*_*max*_. In contrast, males of west origin displayed consistently larger selection estimates than females and the difference in the regression slope, *β*_*ss*_, was much more pronounced. In all cases *β*_*ss*_ was significantly different from zero. The low variance in RS in western sneaker males resulted in low selection estimates. Looking across gender and origin, selection differentials for females of south origin were consistently larger than for females of west origin except for the Bateman gradient (Table 7). In contrast, selection estimates in western males were consistently higher than in southern males, and the resulting *β*_*ss*_ was 1.7 times higher. We refrained from interpreting the analysis on sneaker males as the contribution of specimens of south origin was limited to only two offspring.

**TABLE 7:** Mating system and sexual selection statistics.

		*I*	*I*_s_	*s*	*m*	*β*_ss_	*s*_max_
Origin	South (*n* = 151)	6.902	2.735	0.041	0.123	1.187	1.964
	West (*n* = 167)	10.193	2.483	0.175	0.089	1.794	2.830
South	Females (*n* = 78)	6.999	3.195	0.121	0.384	1.095	1.958
	Nesting males (*n* = 64)	2.052	1.743	−0.280	−0.268	1.022	4.034
	Sneaker males (*n* = 9)	4.5	4.5	0	0	0.889	1.886
West	Females (*n* = 84)	3.843	1.404	−0.100	−0.045	1.369	1.622
	Nesting males (*n* = 59)	16.052	4.692	0.446	0.383	1.730	3.747
	Sneaker males (*n* = 24)	1	1	0.001	−0.114	0.911	0.911

*Note:* The opportunity for selection (*I*), the opportunity for sexual selection (*I*_s_), the standardized selection differential (*s*), the standardized mating differential (*m*), the Bateman gradient (*β*_ss_), the maximum standardized sexual selection differential (*s*_max_). In parenthesis, are the number (*n*) of individuals for each group.

### 5.3 Winter Mortality

**FIGURE 4:**
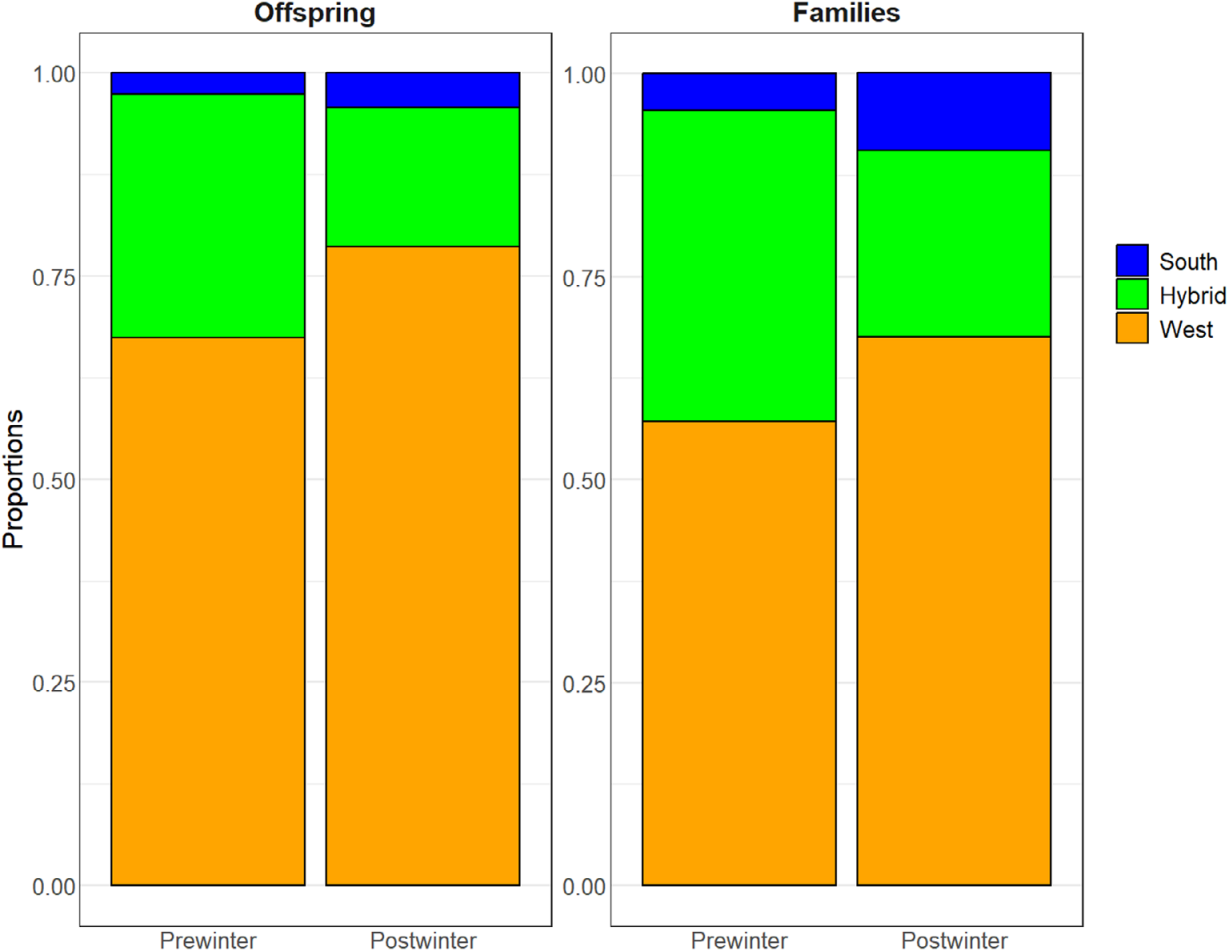
Changes in the proportion of offspring and families of South, Hybrid and West origin observed before (Pre‐winter, retrieved from BG2019) and after (Post‐winter) the harsh winter conditions experienced in the experimental mesocosm basin.

This study shows a drastic shift in family composition after the harsh winter conditions experienced in the mesocosm basin (post‐winter) compared to the situation previously examined with the same cohort of age‐0 juveniles the previous summer (pre‐winter, BG2019; Table 8, Figure 4). While the smaller number of offspring genotyped in the current study resulted in fewer families identified, the parentage assignment revealed an increase of almost 20% in the total number of breeders contributing to the offspring in the current study; hence some families were previously overseen. Such an increase was particularly pronounced among nesting males of both south and west origin as well as among females of west origin (Table 8). Interestingly, despite the significant increase in the number of contributing breeders, a drastic change was observed in the proportions of offspring and families of south, hybrid and west origin (Figure 4). While the proportion of offspring and families of both south and west origin increased by 1.6% and 11.2%, respectively, there was a reduction in the proportion of offspring of hybrid origin by 12.8%. Similarly, the proportion of families of south and west origin increased by 5.0% and 10.4%, respectively; in contrast to the reduction exceeding 15% in the proportion of families of hybrid origin experienced at the end of the experiment.

**TABLE 8:** Parental contribution to the pre‐ and post‐winter offspring.

	Pre‐winter BG2019	Post‐winter this study	Post‐ vs. pre‐winter (change ±)
No. breeders	123 (38.7)	185 (58.2)	62 (+19.5)
No. males per origin	13/39 (17.8/47.0)	31/52 (42.5/62.6)	22/13 (+24.7/+15.6)
No. nesting males per origin	13/22 (20.3/37.3)	29/45 (45.3/76.2)	16/23 (+25/+35.9)
No. sneaker males per origin	0/17 (54.8)	2/17 (22.2/54.8)	2/0 (+22.2/0)
No. females per origin	25/46 (32.0/70.8)	29/63 (37.2/75.0)	4/17 (+5.1/+20.2)
No. families	243	222	−21
Largest family size per origin	6/12/31	1/14/45	−5/+2/+14

*Note:* Number of breeders (% of total *n* = 318), number of males of south/west origin (% of total, *n* = 73 and 83, respectively), number of nesting males of south/west origin (% of total, *n* = 64 and 59, respectively), number of sneaker males of south/west origin (% of total, *n* = 9 and 24, respectively), number of females of south/west origin (% of total, *n* = 78 and 84, respectively), number of families, and largest family size of south/hybrid/west origin. The last column shows the differences (+: increase or −: reduction) between the number and % of individuals obtained in the present study (Post‐winter) from those obtained from the Pre‐winter study (retrieved from BG2019).

## 6 Discussion

### 6.1 Reproductive Success and Mating Success

In this study, we followed up the dynamics of a corkwing wrasse cohort naturally born in a large semi‐natural mesocosm basin (for details, see BG2019) over a 2‐year period to investigate the ecological interactions and potential mechanisms of evolutionary divergence between southern and western populations of a coastal rocky shore fish. In agreement with the previous study (BG2019), our results evidenced significant differences in both mating and reproductive success between the two populations. However, the current analysis performed on the winter survivors suggested unprecedented assortative mating patterns between breeder pairs of southern and western wrasses (*χ* ^2^ = 20.12, *p* < 0.001, Table 5). Our pedigree analyses revealed that an additional contribution of approximately 20% of the breeders and almost half of the families of south origin were unnoticed in BG2019 (Table 8). Two of the newly detected contributors survived the complete experimental period and were one of the 37 parental fish identified when the mesocosm was emptied. Hence, the discrepancy in the results of the assortative mating analyses between the two studies seems to be, at least partly, associated with incomplete sampling in the earlier study. In Norway, corkwing wrasse spawns naturally during late spring to early summer, coinciding with the bloom of phytoplankton, which reduced water visibility and hindered direct observations of mating behaviour and spawning events in the mesocosm. Incomplete observations or partial offspring sampling can obscure patterns and lead to misinterpretation of mate choice and parental contribution, resulting in a major cause for under‐estimation or failure to detect assortative mating (May et al. 2023; Rios Moura et al. 2021; Rolán‐Alvarez et al. 2015). These limitations are compounded by the parental effects on early offspring survival, which can disproportionately remove certain families and ultimately reshape the genetic architecture of the surviving cohort (Alonzo 2010; Myhre et al. 2012; Moginie and Shima 2018). The high natural mortalities of the offspring between the two sampling periods may have further accentuated the differences. These considerations suggest that the apparent discrepancies between our study and BG2019 likely reflect a combination of methodological limitations, environmental constraints, and the inherently complex mating system of the species. In their systematic review, Rios Moura et al. (2021) found that patterns of assortative mating may preset low replicability in space and time in most taxa and advocated for careful interpretation before drawing general conclusions.

Our results suggest that successful mating in corkwing wrasse is primarily driven by local parental origin (Table 6). On the other hand, neither the length of the fish, a phenotypic trait reported to differ significantly between genders and geographical regions (Halvorsen et al. 2016), nor the reproductive behavior of the males (territorial vs. sneaker), an alternative reproductive tactic observed in many species to gain access to mating opportunities (Taborsky 1998; Uglem and Rosenqvist 2002; Oliveira et al. 2008), explains mating success. Reproductive success was significantly higher among males of western origin, in contrast to the poor performance exhibited by sneakers. Overall, western wrasses exhibited higher RRS compared to southern individuals, both within gender (Table 3) and breeding pairs (Table 4).

Non‐random mating limits gene flow and its account is crucial to understand reproductive isolation and speciation processes. Earlier studies in plants (Lowry et al. 2008), insects (Matsubayashi and Katakura 2009), marine snails (Perini et al. 2020) and fish (Lackey and Boughman 2017) have suggested that premating reproductive barriers, such as habitat and sexual isolation, evolve early in divergence and are stronger than postmating barriers. Assortative mating may evolve over time and ongoing gene flow is possible even under strong assortative mating (Lackey and Boughman 2017; Servedio and Boughman 2017; Mattingsdal et al. 2020; May et al. 2023). Schumer et al. (2017) observed that assortative mating in hybrid swordtail fishes *Xiphophorus* was plastic and may disappear under manipulated conditions, and suggested that it may constitute an intermittent and unpredictable barrier to gene flow. Our results support previous studies pointing out towards reproductive isolation and early speciation processes in Scandinavian corkwing wrasses (Blanco Gonzalez et al. 2016; Mattingsdal et al. 2020; Faust et al. 2021). The oceanographic biophysical model implemented on corkwing wrasse suggested a higher degree of retention in the small coastal fjords in southern Norway than in the deeper western fjords (Blanco Gonzalez and Albertsen 2025). Besides significant phenotypic differences in life history traits (Halvorsen et al. 2016), genetic studies evidenced habitat and reproductive isolation between southern and western wrasse populations (Blanco Gonzalez et al. 2016; Mattingsdal et al. 2020). Mattingsdal et al. (2020) detected ongoing asymmetric gene flow between the two populations across a genetic break and suggested recent secondary contact after divergence or negative selection acting against hybrids. Here, family reconstruction confirmed that hybrid offspring experienced strong mortalities during cold winter periods (Figure 4).

Environmental changes and anthropogenic pressures (e.g., fishing and translocations) can affect gene flow, induce phenotypic changes in sexually selected traits, alter mating system patterns and affect the dynamics of the speciation process (Servedio and Boughman 2017; Sbragaglia et al. 2019; Matte et al. 2023). Matte et al. (2023) observed that selective harvesting led to substantial changes in the size‐structure of brook trout *Salvelinus fontinalis* . Under experimental conditions, large zebrafish ( *Danio rerio* ) displayed an evolutionary adaptation in the reproductive performance to offset harvesting regimes (Sbragaglia et al. 2019). The Norwegian corkwing wrasse fishery is size‐ and sex‐selective with the salmon industry particularly interested in large territorial males (Blanco Gonzalez and de Boer 2017). Additionally, millions of wrasses are translocated annually and escapees have been often reported (Blanco Gonzalez and de Boer 2017; Faust et al. 2018, 2021). Based on these two particular features, we anticipate strong anthropogenic‐induced selection pressures affecting the trajectories of wild corkwing wrasse populations in Norway.

## 7 Sexual Selection

Our results evidenced a diverse array of mating behaviors in corkwing wrasse, including monogamy, polygyny, polyandry and polygynandry. Similarly to other fish, corkwing wrasse can adopt alternative reproductive tactics to overcome the variability of mates available and successfully maximize the reproductive output (Emlen and Oring 1977; Avise et al. 2002; Alonzo 2010; Coleman and Jones 2011). Multiple paternity can help to maintain the genetic diversity in wild fish populations (Alonzo and Heckman 2010; Coleman and Jones 2011; Stiver et al. 2018; Svensson and Kvarnemo 2023). The density and social interactions between individuals carrying particular genotypes and phenotypes can alter the strength and direction of sexual selection (Avise et al. 2002; Kokko and Rankin 2006; Aronsen et al. 2013; Alonzo and Servedio 2019). In our mesocosm, western wrasses monopolized the offspring production with almost 80% (393) of the offspring assigned to breeder pairs of western origin and an additional 17% (86) of them of mixed parental origin. This resulted in some large families of western origin and higher standardized variance in RS (i.e., *I*) compared to southern wrasses (Table 7). In contrast, the standardized variance in MS (i.e., *I*_*s*_) was slightly higher in the breeders of southern origin. Estimates of Bateman's gradient, *β*_*ss*_, of translocated western wrasses was 1.5 times steeper than that of the local southern population, suggesting stronger intensity of sexual selection operating in non‐local western individuals. Looking at gender for each origin separately, southern females experienced stronger sexual selection than males, while the opposite was true for the western population. Looking across gender and origin, southern females experienced stronger sexual selection than western females; in contrast to the males, where sexual selection was more pronounced among western individuals. Hence, under the semi‐natural conditions of the mesocosm, corkwing wrasse limitations of mating opportunities appear to be stronger for local southern females and translocated western males. The upper limit of intensity of selection, *s*_*max*_, revealed that intensity of selection on fish length barely accounted for 2% to 12%, which suggests that most of the variation in reproductive success in corkwing wrasse must be attributed to other traits not examined here or emerged as result of stochastic factors (Jones 2009).

Despite wrasses in South and West Norway display significant differences in body sizes (Halvorsen et al. 2016), sexual selection in corkwing wrasse appears not to be exclusively driven by phenotypic differences in the body length of the fish. Uglem and Rosenqvist (2002) observed that larger corkwing wrasse males tend to start nest building earlier and construct larger nests than smaller males; however, in their experiments, they found no correlation of fish size with mating success. Investigating the mating dynamics in another wrasse species, ocellate wrasse *Symphodus ocellatus* , Stiver et al. (2018) observed the engagement of a female in 12 spawning events at different nets within a three‐week period. In gobiids, habitat complexity may relax the strength of sexual selection by affecting the mating behavior of both females and males (Myhre et al. 2012). Brain size and cognitive ability may be also determinant for mate choosiness in sticklebacks (Álvarez‐Quintero et al. 2021). Sexual selection was also highlighted as a major driver shaping fitness variation between local, foreign and their F1 hybrids in a reciprocal transplantation study conducted on brown trout, *Salmo trutta* (Labonne et al. 2021). Hence, in agreement with our findings in corkwing wrasse, selection on traits related to mate choice and competition in parental care species appears to respond to complex social or behavioural interactions within and between sexes (Alonzo and Heckman 2010; Myhre et al. 2012; Bose et al. 2018; Moginie and Shima 2018; Sinopoli et al. 2018; Stiver et al. 2018; Álvarez‐Quintero et al. 2021; Svensson and Kvarnemo 2023).

## 8 Selective Mortality of Hybrid Genotypes

Following up the group of winter survivors of the same cohort examined by BG2019, it was possible to gain insight into natural selection mechanisms acting upon corkwing wrasses during the juvenile stage. We observed major changes in family composition and breeders' contribution between the pre‐wintering and post‐wintering situations, with a significant reduction in the proportion of hybrid offspring (Figure 4). The pedigree analysis revealed that almost 60% of the broodstock introduced in the mesocosm contributed to the offspring during the reproductive season, an increase of almost 20% compared to the pre‐wintering estimates. Among them, the presence of nesting males of both southern and western origin as well as females of western origin was particularly relevant. Additionally, family reconstruction of the post‐wintering survivors disclosed the reduction of 42 (15.3%) families of hybrid origin, in contrast to the increase registered in both south and west families compared to the pre‐winter condition (BG2019). Besides evidencing the abovementioned sample size limitations of the previous study to identify all breeding pairs and characterize the mating system in the mesocosm, current results suggest stronger selective mechanisms acting against hybrid offspring during the juvenile stage. Corkwing wrasse in Norway are at the northern limit of their distribution range and have been reported to experience high mortalities when seawater temperatures fall below 4°C (Bjelland et al. 1996). During the experimental period, wrasses experienced one of the coldest winters recorded over the last decades (www.imr.no/forskning/forskningsdata/temperatur/flodevigen), and some sections of the upper layer of the mesocosm appeared frozen during the coldest days (Blanco Gonzalez, pers. observation). Therefore, the changes in family composition observed between the juveniles analyzed by BG2019 and the present study may be due to stronger selective winter mortality acting against hybrid genotypes.

These results support the hypothesis that negative selection against hybrid individuals contributes to preserve the strong genetic divergence between southern and western corkwing wrasse populations despite ongoing gene flow across a genetic break in southwestern Norway (Mattingsdal et al. 2020). Moreover, current findings suggest that artificial translocations between southern and western origin corkwing wrasses may overcome reproductive isolation imposed by natural processes (Blanco Gonzalez et al. 2016, 2019; Faust et al. 2018, 2021; Mattingsdal et al. 2020). It is noteworthy, though, that despite the large size of the mesocosm, the seminatural experimental setup may not fully mirror the natural processes encountered in the wild. It is not possible to neglect putative negative density‐dependent effects on mating and offspring survival due to limitations of space and/or food (Kokko and Rankin 2006; Aronsen et al. 2013; Stiver et al. 2018). Hence, extrapolation of these results to the wild demands some caution.

## 9 Effective Population Size and Implications for Aquaculture‐Based Fisheries Enhancement Systems

The effective population size, or as in the present case of a single mating season—the effective number of breeders (*Nb*)—is an important concept in management of genetic diversity within wild and artificially reared fish. Our finding of small effective to census ratios (0.0629 for the combined population) means that offspring will be much more closely related than expected from the relatively large number of breeders used (318). This increased relatedness would have resulted in increased inbreeding and loss of genetic diversity in future generations had the offspring been allowed to reproduce. While our mesocosm may fail to reproduce natural conditions, our findings are highly relevant for artificial rearing of corkwing wrasses and other species as cleaner fish for the salmon industry. Hence, such rearing is likely to produce large numbers of related offspring, with potentially negative impact on natural populations, should they escape the salmon pens and reproduce with wild conspecifics.

Inadvertent loss of genetic diversity is arguably the main concern for intentional juvenile releases in marine restoration and aquaculture‐based fisheries enhancement programs (Taniguchi 2003; Araki and Schmidt 2010; Laikre et al. 2010). These programs advocate for using a large number of wild local specimens as breeders and their regular replacement with newly collected wild individuals as fundamental procedures to preserve the genetic integrity of local populations (Taniguchi 2003; Blanco Gonzalez et al. 2010). Additionally, it is advised to rear the juveniles under near‐natural conditions and minimize their time in captivity to avoid any putative inadvertent reduction in fitness. However, the difficulty to follow up the fate of the released organisms throughout their entire life may partly justify why lifespan reproductive success and long‐term genetic monitoring in fish are mainly from salmonids (see reviews by Araki and Schmidt 2010; Laikre et al. 2010) with scarce knowledge on iteroparous marine species (Jeong et al. 2007; Blanco Gonzalez, Nagasawa, and Umino 2008; Blanco Gonzalez, Umino, and Nagasawa 2008; Blanco Gonzalez et al. 2015; Kitada et al. 2019). Genetic monitoring of marine fish enhancement programs has been usually restricted to estimates of the genetic inheritance in small offspring before release (Araki and Schmidt 2010). Minimizing the time in captivity can also help to reduce putative risks of unintentional domestication and contribute to make these programs economically viable (Taniguchi 2003; Berejikian et al. 2009; Araki and Schmidt 2010). However, although such analyses may provide a good first overview of reproductive behavior, family composition and genetic architecture of the offspring for release, they may limit the analytical power to resolve subtle assortative mating patterns and the putative genetic inheritance to future generations, as observed in corkwing wrasse here.

Besides inferences on the number of survivors reaching maturity and their genetic inheritance, extending the experimental period of the offspring in the mesocosm unveiled high mortalities without any putative predator. This suggests that the low recapture rates reported in many stocking programs may be partly explained by mortalities associated with environmental selection (i.e., temperature) and not necessarily to vulnerability to predation as previously suggested in many studies (see review by Araki and Schmidt 2010). This information can be crucial to optimize the release strategies to maximize survival. As pointed out earlier, our results are based on a seminatural experimental setup which may not fully mirror the natural reproductive behavior of the species in the wild. Additionally, we followed a single fish cohort, thus not representing the overall contribution of the broodstock of this iteroparous fish species. Yet, our results encourage the implementation of long‐term genetic monitoring as routine components to unveil selective mechanisms, minimize putative risk of inbreeding, and contribute to preserve the genetic resources in marine restoration and aquaculture‐based fisheries enhancement programs.

## Funding

This work was supported by the Norwegian Research Council (Havkyst Grant No. 234328 and Marinforsk Grant No. 243894); the EU Interreg program Øresund‐Kattegat‐Skagerrak (Project “BioBlueClimate” to P.E.J.); and the FCT—Foundation for Science and Technology (Grant Nos. MARE/UIDB/MAR/04292/2020, MARE/UIDP/04292/2020, and LA/P/0069/2020 to J.I.R., granted to MARE/ARNET).

## Conflicts of Interest

The authors declare no conflicts of interest.

## Supporting information


**Table S1:** Summary statistics of genetic variability at 10 microsatellite markers for the breeders (south and west origin) and offspring (south, hybrid and west) corkwing wrasse genotyped in this study. Bold values indicate significant *p* values at 5% level after the false discovery rate approach (Benjamini and Hochberg 1995).
**Table S2:** Pairwise comparisons among breeders and offspring corkwing wrasse at 10 microsatellite markers. Values below the diagonal are *F*_ST_ estimates for all sample pairs and above the diagonal are the corresponding levels of significance. In bold are highlighted those estimates remaining significant at 5% after the false discovery rate approach (Benjamini and Yekutieli 2001).

**Figure S1:** Principal Components Analysis (PCA) illustrating the genetic differentiation among breeders and offspring corkwing wrasse based on the two main principal components. Abbreviation for the samples: BrS = Breeders south (blue); BrW = Breeders west (orange); OffS = Offspring south (light blue); OffH = Offspring hybrid (green); OffW = Offspring west (light grey).

**Figure S2:** Number of mating pairs by breeder according to broodstock origin (South, A–D and West E–H) and sex (Females, A–C and E–G, and Males D and H). The numbers of mates for females are shown combining dominant and sneaker males (A, E), only considering dominant males (B, F) and only considering sneaker males (C, G). Blue (South) and orange (West) colors represent the origin of the mating pair. Note the difference in the scale of the y‐axis.

## Data Availability

The dataset to replicate the analyses conducted in this study is available on DataverseNO public repository (https://doi.org/10.18710/VPXHVP).
