# Supplementary material for: Selection Against Hybrids Maintains Genetic Divergence Between Populations of a Coastal Cleaner Fish Translocated Across a Genetic Break
Source: Evol Appl. 2026 Mar 15;19(3):e70214. doi: 10.1111/eva.70214 (PMC13093811; doi:10.1111/eva.70214)
Supplement: Supplementary file 1 — Table S1: Summary statistics of genetic variability at 10 microsatellite markers for the breeders (south and west origin) and offspring (south, hybrid and west) corkwing wrasse genotyped in this study. Bold values indicate significant p values at 5% level after the false discovery rate approach (Benjamini and Hochberg 1995). Table S2: Pairwise comparisons among breeders and offspring corkwing wrasse at 10 microsatellite markers. Values below the diagonal are F ST estimates for all sample pairs and above the diagonal are the corresponding levels of significance. In bold are highlighted those estimates remaining significant at 5% after the false discovery rate approach (Benjamini and Yekutieli 2001). [file EVA-19-e70214-s003.docx]

**Supplementary Material**

**TABLE S1**. Summary statistics of genetic variability at 10 microsatellite markers for the breeders (south and west origin) and offspring (south, hybrid and west) corkwing wrasse genotyped in this study. Bold values indicate significant *p* values at 5% level after the false discovery rate approach (Benjamini & Hochberg, 1995).

Abbreviation: *A*: number of alleles; *H*_T_: gene diversity (expected heterozygosity) in the total material; *F*_IS_: inbreeding coefficient representing deviations from Hardy–Weinberg proportions; *F*_ST_: level of genetic differentiation; *PIC*: polymorphic information content; *I*: probability of identity index.

|  | *Locus* | SMD121 | SMA11 | SMA103 | SMD131 | SMD110 | SMD112 | SMB11 | SMC8 | SMB101 | SMC5 | Overall |
| --- | --- | --- | --- | --- | --- | --- | --- | --- | --- | --- | --- | --- |
| TOTAL | *A* | 30 | 5 | 4 | 16 | 11 | 14 | 26 | 15 | 40 | 14 | 175 |
| (n=819) | *H*_T_ | 0.862 | 0.519 | 0.734 | 0.567 | 0.637 | 0.667 | 0.802 | 0.839 | 0.908 | 0.776 | 0.731 |
|  | *F*_IS_ | **-0.034** | **0.027** | **-0.022** | -0.018 | -0.023 | -0.079 | **-0.048** | **-0.057** | **-0.036** | -0.057 | **-0.038** |
|  | *F*_ST_ | **0.076** | **0.070** | **0.081** | **0.036** | 0.004 | **0.044** | **0.129** | **0.086** | **0.027** | **0.012** | **0.059** |
|  | *PIC* | 0.869 | 0.444 | 0.652 | 0.535 | 0.555 | 0.706 | 0.858 | 0.814 | 0.883 | 0.734 | 0.705 |
|  | *I* | 0.024 | 0.305 | 0.140 | 0.222 | 0.210 | 0.097 | 0.029 | 0.047 | 0.020 | 0.087 | 1.1E-11 |

**TABLE S2**. Pairwise comparisons among breeders and offspring corkwing wrasse at 10 microsatellite markers. Values below the diagonal are *F*_ST_ estimates for all sample pairs and above the diagonal are the corresponding levels of significance. In bold are highlighted those estimates remaining significant at 5 % after the false discovery rate approach (Benjamini & Yekutieli, 2001).

|  | **Breeder south** | **Breeder west** | **Offspring south** | **Offspring hybrid** | **Offspring west** |
| --- | --- | --- | --- | --- | --- |
| **Breeder south** |  | **+** | **+** | **+** | **+** |
| **Breeder west** | 0.094 |  | **+** | **+** | - |
| **Offspring south** | -0.001 | 0.081 |  | **+** | **+** |
| **Offspring hybrid** | 0.040 | 0.029 | 0.030 |  | **+** |
| **Offspring west** | 0.118 | 0.016 | 0.100 | 0.029 |  |

**Supplementary figures**

**FIGURE S1**. Principal Components Analysis (PCA) illustrating the genetic differentiation among breeders and offspring corkwing wrasse based on the two main principal components. Abbreviation for the samples: BrS = Breeders south (blue); BrW = Breeders west (orange); OffS = Offspring south (light blue); OffH = Offspring hybrid (green); OffW = Offspring west (light grey).

**FIGURE S2**. Number of mating pairs by breeder according to broodstock origin (South, A-D and West E-H) and sex (Females, A-C and E-G, and Males D and H). The numbers of mates for females are shown combining dominant and sneaker males (A&E), only considering dominant males (B&F) and only considering sneaker males (C&G). Blue (South) and orange (West) colors represent the origin of the mating pair. Note the difference in the scale of the y-axis.
