# Supplementary figures and images for: Selection Against Hybrids Maintains Genetic Divergence Between Populations of a Coastal Cleaner Fish Translocated Across a Genetic Break
Source: Evol Appl. 2026 Mar 15;19(3):e70214. doi: 10.1111/eva.70214 (PMC13093811; doi:10.1111/eva.70214)

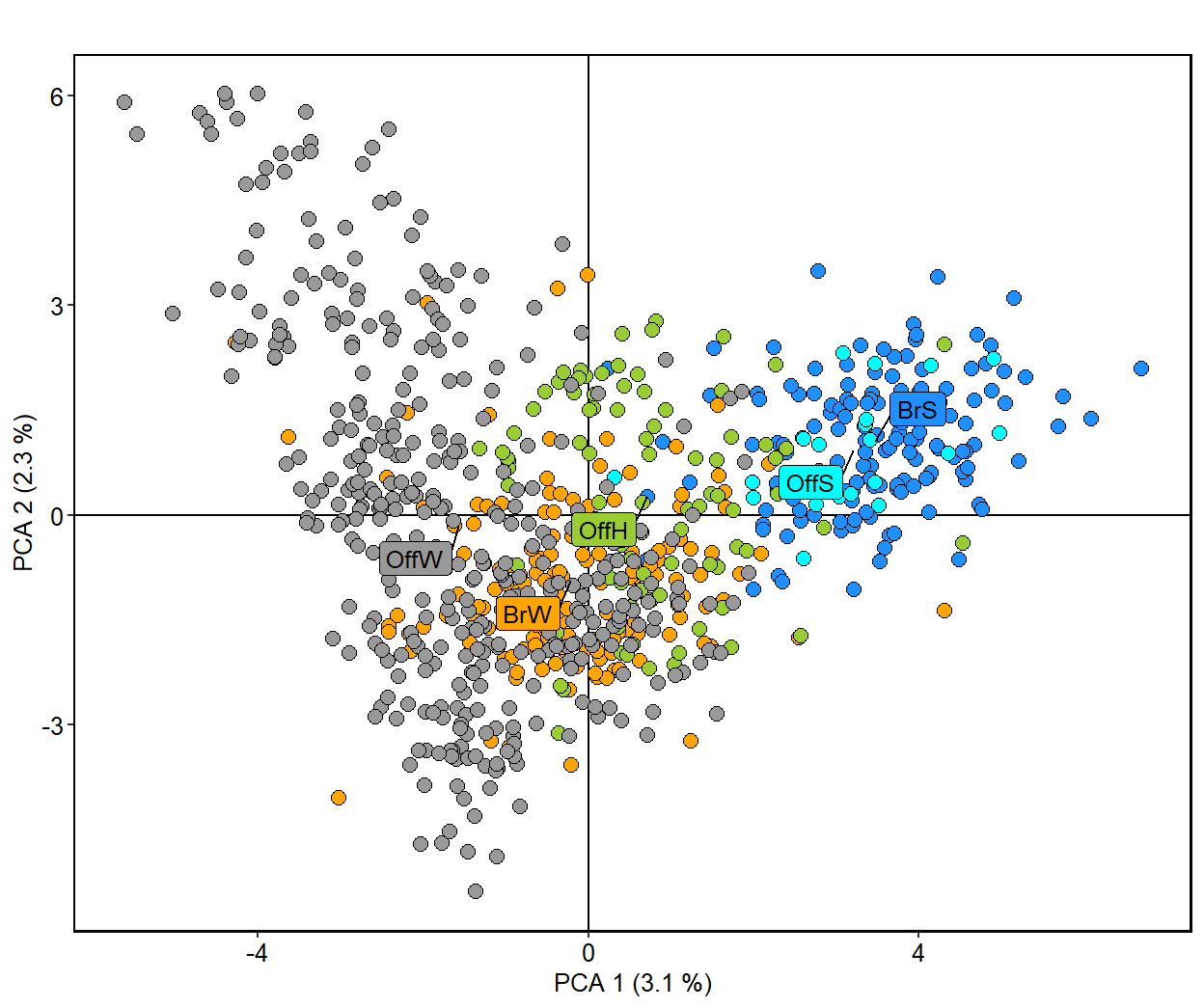

Supplement: Supplementary file 2 — Figure S1: Principal Components Analysis (PCA) illustrating the genetic differentiation among breeders and offspring corkwing wrasse based on the two main principal components. Abbreviation for the samples: BrS = Breeders south (blue); BrW = Breeders west (orange); OffS = Offspring south (light blue); OffH = Offspring hybrid (green); OffW = Offspring west (light grey). [file EVA-19-e70214-s001.tiff]

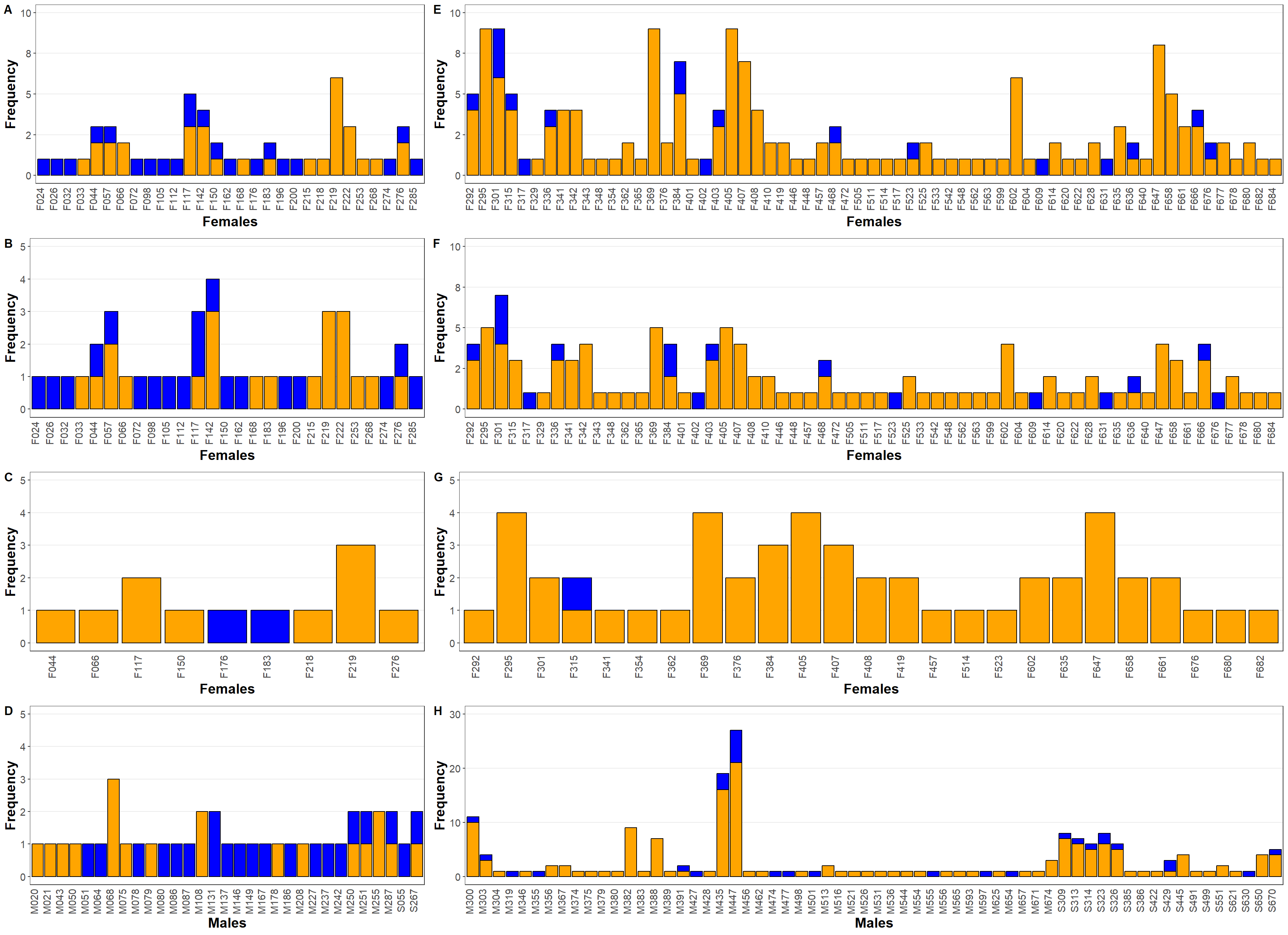

Supplement: Supplementary file 3 — Figure S2: Number of mating pairs by breeder according to broodstock origin (South, A–D and West E–H) and sex (Females, A–C and E–G, and Males D and H). The numbers of mates for females are shown combining dominant and sneaker males (A, E), only considering dominant males (B, F) and only considering sneaker males (C, G). Blue (South) and orange (West) colors represent the origin of the mating pair. Note the difference in the scale of the y‐axis. [file EVA-19-e70214-s002.tiff]
